# NLRP12‐PANoptosome in haemolytic, infectious and inflammatory diseases

**DOI:** 10.1002/ctm2.1409

**Published:** 2023-09-12

**Authors:** Rebecca E. Tweedell, Thirumala‐Devi Kanneganti

**Affiliations:** ^1^ Department of Immunology St. Jude Children's Research Hospital Memphis Tennessee USA

1

Invading pathogens and homeostatic perturbations activate the innate immune system as the body's first line of defense. Innate immunity is driven by pattern recognition receptors (PRRs),^1^ which can activate by: 1) acting as direct receptors that bind specific pathogen‐associated molecular patterns (PAMPs) or endogenous damage‐associated molecular patterns (DAMPs), or 2) sensing and responding to homeostatic perturbations caused by PAMPs and DAMPs. In both cases, PRR activation drives downstream signalling to induce a broader immune response, inflammation, and regulated cell death. The fields of immunology and cellular biology have a long‐standing interest in the NOD‐like receptor (NLR) family of innate immune cytosolic PRRs due to their diverse roles in cell death, inflammation and disease. However, despite more than 20 years of study, relatively little is known about what specific triggers activate many NLRs and how this affects pathology.

One of the enigmatic NLRs is NLRP12. During infections, such as *Yersinia pestis* or *Plasmodium chabaudi*, NLRP12 was thought to act as a sensor to form a multiprotein complex called the inflammasome,^2^ which contains ASC and caspase‐1 and drives the cell death pathway pyroptosis. However, NLRP12 was also shown to have inflammasome‐independent, anti‐inflammatory functions during *Salmonella* infection,^3^ and loss of NLRP12 in mice increases susceptibility to colon inflammation, colorectal tumor development and atypical neuroinflammation.^4^, ^5^, ^6^ Over the years, NLRP12 has remained enigmatic, with both pro‐ and anti‐inflammatory functions reported, and a two‐decades‐long search failed to identify its specific trigger and signalling pathways.

A recent study solved the mystery of NLRP12 by using a unique approach that identified the trigger combinations, instead of single PAMPs or DAMPs, that mimic physiologically relevant conditions and induce NLRP12‐dependent inflammatory cell death, PANoptosis (Figure 1).^7^ PANoptosis is a unique innate immune, lytic and inflammatory cell death pathway driven by caspases and RIPKs that is regulated by the multiprotein PANoptosome complex. The concept of PANoptosis is similar to that of pyroptosis driven by inflammatory caspases in inflammasomes to induce lytic cell death except for the unique composition of the PANoptosome complex, which differentiates it from inflammasomes.

**FIGURE 1:**
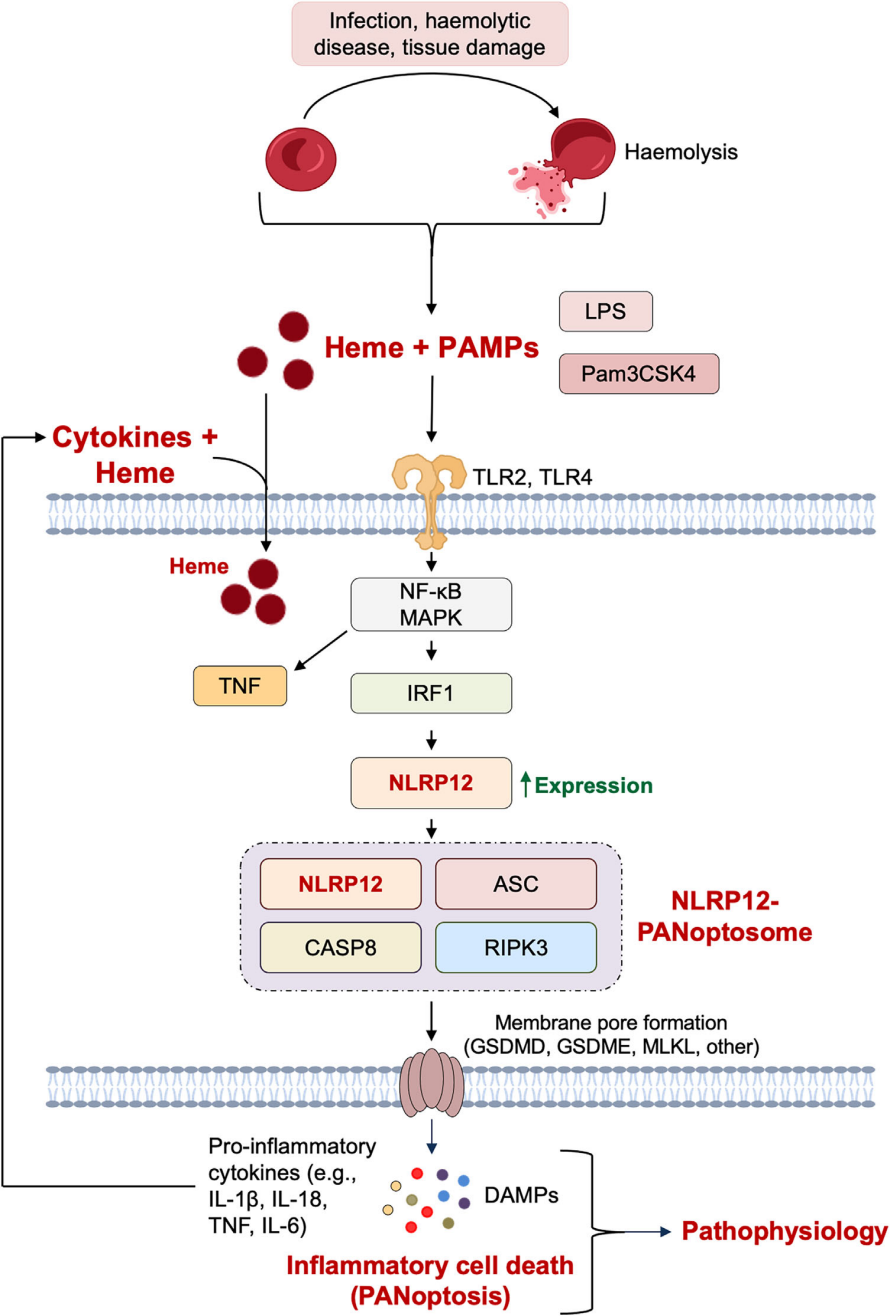
NLRP12‐mediated PANoptosis drives cytokine and damage‐associated molecular pattern (DAMP) release in pathophysiology. Haemolysis results in the release of heme into the bloodstream, which can synergize with pathogen‐associated molecular patterns (PAMPs), such as lipopolysaccharide (LPS) and Pam3CSK4, that are present during disease. Sensing of these combinations through TLR2 and TLR4 leads to nuclear factor kappa B (NF‐κB) and MAPK signalling, which induce pro‐inflammatory cytokine production, such as tumour necrosis factor (TNF), and IRF1‐mediated upregulation of the cytosolic innate immune sensor NLRP12. These pathways allow NLRP12 to form the PANoptosome and induce inflammatory cell death, PANoptosis. PANoptosis releases more pro‐inflammatory cytokines and DAMPs, which can drive further pathophysiology in the disease.

This study showed that heme, combined with specific components of infection or cellular damage, can activate NLRP12 to drive PANoptosis and pathology in disease (Figure 1).^7^ Haemolysis occurs in many conditions, including haemolytic diseases like malaria and sickle cell disease, but also infections such as severe acute respiratory syndrome coronavirus 2, influenza and bacterial pneumonia. This haemolysis results in the accumulation of free heme in the bloodstream, which is associated with inflammation and organ damage. NLRP12 was highly expressed in patients with these infectious and inflammatory diseases,^7^ pointing to a potential role for this sensor in pathophysiology. Previous studies showed NLRP3 is activated by heme alone in vitro^8^, ^9^; however, under physiological conditions, PAMPs and DAMPs are present simultaneously, which can have distinct impacts on innate immune activation. Therefore, to take a new approach and more closely mimic physiological conditions, heme was combined with microbial components, such as the PAMP LPS or Pam3CSK4, or the pro‐inflammatory cytokine tumour necrosis factor (TNF) in this study. These combinations activated robust inflammatory cell death that was dependent on NLRP12 and characterised by activation of specific cell death molecules, including caspase‐1, GSDME, caspase‐8, caspase‐3, caspase‐7 and pMLKL, an activation signature consistent with PANoptosis (Figure 1).^7^ While NLRP3 contributed to inflammasome and caspase‐1 activation in this context, it had no effect on cell death or the activation of other PANoptotic molecules.^7^ Instead, NLRP12 formed a PANoptosome complex with ASC, caspase‐8 and RIPK3, and potentially other molecules that remain to be characterised, to drive PANoptosis.^7^

The formation of the NLRP12‐PANoptosome in response to heme plus PAMPs or TNF suggests a critical role for NLRP12‐mediated PANoptosis in innate immune responses to conditions where free heme is released, including haemolytic diseases, infections, inflammatory conditions and some cancers. Furthermore, deletion of *Nlrp12* significantly reduced pathology and lethality in a murine model of haemolytic disease,^7^ providing further physiological relevance and evidence for NLRP12‐mediated PANoptosis driving inflammation and disease. Previous research has also linked genetic mutations in *NLRP12* to periodic fever syndromes, suggesting there may also be a central role for PANoptosis in these conditions; this requires further study.

Given the clear role established in this study for NLRP12‐dependent inflammatory cell death in haemolytic pathophysiology, it is essential to tightly regulate NLRP12 activation to prevent aberrant signalling. Indeed, the NLRP12‐PANoptosome was regulated through NLRP12 expression, suggesting that NLRP12 is not directly binding to heme or PAMPs for sensing but is instead sensing the associated homeostatic perturbations. Under basal conditions, there was minimal NLRP12 expression, but stimulation with heme plus PAMPs induced robust expression. Similarly, heme plus TNF also induced NLRP12 expression,^7^ suggesting a potential role for cytokines in this process. However, TNF alone was not sufficient for NLRP12 expression,^7^ and TNF likely has a synergistic role here. In response to innate immune stimulation, TNF is induced downstream of toll‐like receptor (TLR) and nuclear factor kappa B (NF‐κB) signalling. Indeed, TLR2 and TLR4 were critical in the regulation of NLRP12 expression,^7^ suggesting that TLR signalling has a predominant role in driving NLRP12‐mediated PANoptosis. Downstream of TLR2 and TLR4, IRF1 controlled the expression of NLRP12 to regulate PANoptosis.^7^ IRF1 has now been identified as a key regulator of multiple PANoptosomes, including the ZBP1‐, AIM2‐, RIPK1‐ and NLRP12‐PANoptosomes,^7^, ^10^ and its role as a transcription factor to control sensor expression likely contributes to this conserved function.

After the two‐decades‐long search for the trigger of NLRP12, this study not only identified the trigger but also NLRP12's novel role in inducing a unique inflammatory cell death pathway, PANoptosis. These findings establish the mechanism of heme‐mediated pathology through NLRP12 expression and multiprotein complex formation. Given the central role of innate immunity in detecting and counteracting infections and sterile insults, it is fundamental to our understanding of health and disease to identify the functions of innate immune sensors to develop more effective treatments against infectious and inflammatory diseases. Now that NLRP12's regulation and function in inflammatory cell death have been identified in this study,^7^ potential therapies can be developed to target NLRP12 or molecules in its regulatory pathway to prevent cell death and inflammation in diverse infections and inflammatory diseases.

## CONFLICT OF INTEREST STATEMENT

St. Jude Children's Research Hospital filed a provisional patent application on methods for modulating NLRP12, listing Thirumala‐Devi Kanneganti as an inventor (serial no. 63/422,601 and 63/501,430).
